# Efficacy and safety of native type II collagen in modulating knee osteoarthritis symptoms: a randomised, double-blind, placebo-controlled trial

**DOI:** 10.1186/s40634-022-00559-8

**Published:** 2022-12-23

**Authors:** Cheng Luo, Weike Su, Ying Song, Shalini Srivastava

**Affiliations:** 1 Research and Development, Jiaxing Hengjie Biopharmaceutical Co. Ltd, No.20 Tongyi Road, Xinfeng Industrial Park, Jiaxing, 314005 Zhejiang China; 2 Department of Pharmacology, Zhejiang University of Technology, Hangzhou, 310023 Zhejiang China; 3 Clinical Development, Vedic Lifesciences Pvt. Ltd, 118, Morya House, Off New Link Road, Andheri (West), Mumbai, 400053 Maharashtra India

**Keywords:** Undenatured collagen type II, Native type II collagen, Glucosamine hydrochloride, chondroitin sulfate, WOMAC, Knee osteoarthritis

## Abstract

**Purpose:**

Knee osteoarthritis (OA) is the most common form of clinical arthritis in middle-aged and older individuals. Undenatured or native type II (TII) collagen derived from the chicken sternum has a good therapeutic effect on relieving severe pain of OA. Hence, the present study aimed to investigate the efficacy and safety of TII collagen (Native CT-II®) in individuals with knee OA.

**Methods:**

We conducted a 12-week randomised, double-blind, placebo-controlled, parallel-group study on 101 participants aged 40–65 years with knee OA. The participants were randomised to receive either TII collagen, glucosamine hydrochloride + chondroitin sulfate (G + C) or a placebo. The primary outcome was an improvement in the joint health of the participants assessed using the Western Ontario and McMaster Universities Osteoarthritis Index (WOMAC) compared to G + C and placebo.

**Results:**

Compared with the placebo group (*n *= 27), the TII collagen group (*n *= 29) and G + C group (*n *= 29) significantly improved the overall joint health measured by the change in WOMAC total score (week 12: TII collagen = -32.47 ± 19.51 and G + C = -33.74 ± 24.64 vs. placebo = -13.84 ± 17.61; p < 0.05) and relieved knee joint pain (week 12: TII collagen = -5.69 ± 3.66 and G + C = -6.03 ± 4.72 vs. placebo = -2.71 ± 3.95; p < 0.05). The statistically significant effect was observed as early as 4 weeks after the investigational product administration. Additionally, the TII collagen was more effective in improving the quality of life than the G + C.

**Conclusion:**

TII collagen not only has a significantly better effect and high safety profile for OA but also improves the quality of life of patients.

**Level of Evidence:**

Level 1 – Randomized Controlled Trial.

**Trial registration:**

ClinicalTrials.gov Identifier: NCT04470336; First submitted date: July 08, 2020; First posted date: July 14, 2020.

## Introduction

Osteoarthritis (OA), also known as degenerative joint disease, is typically the result of wear and tear and progressive loss of articular cartilage and is the most diagnosed form of arthritis, with its prevalence increasing with age, as well as with external factors such as obesity [1]. The current global prevalence of OA in the knee has been estimated to be 22.9% in adults aged 40 years and above [2]. With such high prevalence, the therapeutic market size for OA is increasing rapidly and is projected to reach USD 11.0 billion by 2025 [3]. Nonsteroidal anti-inflammatory drugs (NSAIDs), physical exercise, intra-articular corticosteroids, and hyaluronic acid injections have been widely used as the first line of defence against symptomatic OA. Most of these treatment options may aid in alleviating pain and disability but do not alter the disease progression. Simultaneously, it has been reported that usage of some of these agents can lead to comorbidities and increased mortality [1, 4]. Nutritional supplements derived from natural ingredients have been historically used as an aid to improve joint health [5]. Glycosaminoglycan-based nutraceuticals such as chondroitin sulfate and glucosamine hydrochloride have been extensively studied for their efficacy against OA as they are the vital components of the extracellular matrix and synovial fluid [6, 7]. Preclinical evidence suggests that these supplements protect the joints against "wear and tear" while stimulating cartilage regeneration [8, 9]. Numerous clinical trials have proven their effect on joint pain and have demonstrated them to be a safer and more tolerable option than NSAIDs [10–13]. However, the therapeutic dosage of these products is stated to be at least 1200 mg, which could pose significant compliance challenges [11].

Although adjuvant methods for OA treatment can significantly relieve the pain of OA, they have disadvantages such as low potency, low tolerability, and a large dosage. The consumption of various forms of collagen, such as undenatured or native type II (TII) collagen, has also been studied for their potential benefits during OA management in pre-clinical and clinical studies, demonstrating positive results with substantially lower therapeutic doses [14–17]. The aim of the study was primarily to determine the effect of TII collagen (Native CT-II®) derived from the chicken sternum on osteoarthritis specific measures as compared to the placebo as well as glucosamine hydrochloride + chondroitin sulfate (G + C) and at the same time assess the safety of the product. It was hypothesised that TII collagen would demonstrate not only statistically better reduction in the symptoms of OA of the knee, but also the reduction will be clinically meaningful.

## Materials and methods

### Study design

A 12-week, randomised, double-blind, placebo-controlled, parallel-group study was conceptualised to evaluate the efficacy and tolerability of TII collagen in patients with knee OA. Five hospitals were selected for the clinical study, 4 in Mumbai and 1 in Varanasi, India. The investigators explained to all the participants about the objectives, procedures, risks, and benefits involved in the study. Only participants who gave written consent voluntarily were recruited into the study. The study results have been reported as per the Consolidated Standards of Reporting Trials statement.

### Participants

Adults of either sex complaining of knee pain with radiographical evidence of OA were recruited for the study. Individuals with other joint pathologies, such as rheumatoid arthritis and gout, were excluded from the trial. Additional inclusion and exclusion criteria for the study are provided in Table 1.

**Table 1:** Main inclusion and exclusion criteria for the study

Criteria	Basic conditions
**Inclusion criteria**	1. Age ≥ 40 to ≤ 65 years
	2. BMI ≥ 18.5 and ≤ 29.9 kg/m^2^
	3. Non-vegetarians
	4. VAS score ≥ 60 mm for knee joint pain
	5. Willing to abstain from food containing Type II collagen from cartilage 48 h before all assessment visits
**Exclusion criteria**	1. FBG > 125 mg/dL
	2. SBP ≥ 140 mmHg and/or DBP ≥ 90 mmHg
	3. Radiographic evidence of Grade I or Grade IV OA based on the Kellgren and Lawrence criteria for osteoarthritis
	4. Any history of trauma, fractures, or surgery to the index joint 5. History of use of corticosteroids (oral or parenteral) and hyaluronic acid (intra-articular) for the last three days
	6. Usage of local and/or systemic analgesics

Abbreviations: *BMI* Body mass index, *DBP* Diastolic blood pressure, *FBG* Fasting blood glucose, *OA* Osteoarthritis, *SBP* Systolic blood pressure, *VAS* Visual analogue scale

### Interventions

The investigational product (IP), TII collagen, contains undenatured/native type II collagen derived from the chicken sternum, as described in Table 2. Previous studies have demonstrated that consuming undenatured type II collagen in small doses effectively improves joint health [18]. The IP, G + C, and placebo capsules were manufactured in a Good Manufacturing Practices compliant facility and were matched for size, shape, colour, and texture and packed in identical plastic bottles with similar labels to preserve the blinding. All the participants were instructed to consume 3 capsules post-breakfast and 3 capsules post-dinner, summing up to a daily dose of 2700 mg. Acetaminophen at a maximum dose of 1000 mg/day was allowed as a rescue medication during the study. However, participants had to abstain from its consumption for 48 h before any study visit.

**Table 2:** IP details (Daily dose)

Active ingredients	TII collagen	G + C	Placebo
Undenatured type II collagen	40 mg	-	-
Glucosamine hydrochloride	-	1500 mg	-
Chondroitin sulfate	-	1200 mg	-
D-glucose	2660 mg	-	2700 mg
Total weight	2700 mg		
Dosage form	White opaque capsule		

Abbreviation: *G + C* Glucosamine hydrochloride and Chondroitin sulfate

### Study conduct

The first participant's first visit was conducted on July 10, 2020, while the last participant's last visit was conducted on April 12, 2021. On the screening visit, prospective participants were evaluated for the pre-defined eligibility criteria. Blood samples were collected to estimate fasting blood glucose levels (colourimetric/spectrophotometric estimation using glucose oxidase peroxidase method). Participants who fulfilled the eligibility criteria were enrolled in the study and instructed to report for baseline assessment. Before randomisation, each participant had to comply with a 7-day placebo run-in period to identify placebo responders. Only participants with a Western Ontario and McMaster Universities Osteoarthritis Index (WOMAC) score ≥ 75, no decrease in pain visual analogue scale (VAS) score compared to screening, and 80% compliance during the run-in phase were randomised for the study. Block randomisation using a block of 6 was performed using the StatsDirect Software version 3.1.17. The random allocation of the IP was performed using the interactive web response system on Clindox by the research staff not directly involved in the study. The participants were randomised in a ratio of 1:1:1 to receive the study product, comparator, or placebo as per the Interactive Web Response System assignment. The blinding for the products was done using the blinding codes, which were secured in tamper-evident sealed envelopes. The participants, investigators, and the research staff directly involved in the study were blinded to the treatment allocation.

The participants were asked to report for the follow-up assessment visits at the end of week 4, 8, and 12 from the randomisation day. Concomitant medication intake was recorded at each visit. Treatment compliance and rescue medication usage were ascertained for each participant through an IP diary to be filled by each participant throughout the study. A record of the dispensed and returned medication was also maintained to confirm treatment compliance at each visit.

### Outcome variables

#### Primary outcome variables

The WOMAC, a participant-administered validated instrument for assessing joint health, was used as a primary outcome variable. The scale has been widely used in clinical trials and consists of 3 subscales comprising 24 questions. Each question was assessed on a Likert-based response rated from 0 to 4 points (0 indicates "no pain" and 4 "extreme pain"). Higher scores indicate a worse clinical condition [19]. To evaluate the efficacy of the IP, the change in the WOMAC total score from baseline to end of week 4, 8, and 12 was compared with that of the placebo.

#### Secondary outcome variables

The secondary outcome of the study consisted of a change in the WOMAC subscale of pain (WOMAC-P; scale of 0–4; averaged response of 5 questions), stiffness (WOMAC-S; scale of 0–4; averaged response for 2 questions), and physical function (WOMAC-PF; scale of 0–4; averaged response for 17 questions) at the end of week 4, 8, and 12 from baseline [20]. Other measures performed included health-related quality of life as evaluated by a validated EQ-5D-5L questionnaire consisting of 5 domains – mobility, self-care, usual activities, pain/discomfort, and anxiety/depression using 5 response levels – no problems, slight problems, moderate problems, severe problems, and extreme problems [21]. The quality of life of the participants was assessed at baseline and end of the study visit (week 12).

#### Safety variables

The pulse and blood pressure were measured at the baseline and end of the study visits. Also, blood samples were collected during these visits to monitor the serum biochemical markers for liver and kidney function, including serum aspartate aminotransferase (AST), alanine aminotransferase (ALT), alkaline phosphatase (ALP), and creatinine. The serum AST and AST levels were analysed using the NADH (without P-5’-P) methodology. Colourimetric and electrochemiluminescence immunoassay techniques were used for ALP and creatinine quantification, respectively. Furthermore, any adverse events and serious adverse events were to be monitored and reported to the investigator. The adverse events were recorded in the source document and the appropriate adverse event module in the electronic-case record form.

### Statistical analysis

The sample size used for the study (*n* = 30 per arm) was similar to that used by Braham et al. [22], to demonstrate efficacy of 12 weeks of glucosamine supplementation in individuals with constant knee pain. Crowley et al. [14] successfully used same number of participants to demonstrate efficacy of 90 days administration of undenatured type II collagen in osteoarthritis of the knee.

With the assumption of approximately 20% of dropouts and withdrawals during the study, 117 participants needed to be recruited for the study. The primary and secondary efficacy variables were summarised descriptively using mean and standard deviation. The type I error probability associated with the null hypothesis test was set at 0.05. The primary efficacy outcome was analysed for the modified intention-to-treat (mITT) population, defined as participants who were randomised in the study and had at least completed the study visit at the end of week 4. The last observation carried forward method was used for missing data imputation. The baseline parameters were analysed using the Chi-square and analysis of variance (ANOVA) tests. The efficacy and safety parameters were analysed by analysis of covariance (ANCOVA) test for between-group comparisons (with treatment as a factor and baseline as a covariate) and a paired t-test to calculate the statistical significance from the baseline within the group. All the statistical analyses were performed using R Ver. 4.0.5 (R Foundation for Statistical Computing, Vienna, Austria. https://www.R-project.org/) and XLSTAT Ver. 2021.3.1 (Statistical and data analysis solution. New York, USA. https://www.xlstat.com./).

## Results

### Participant disposition

**Fig. 1:**
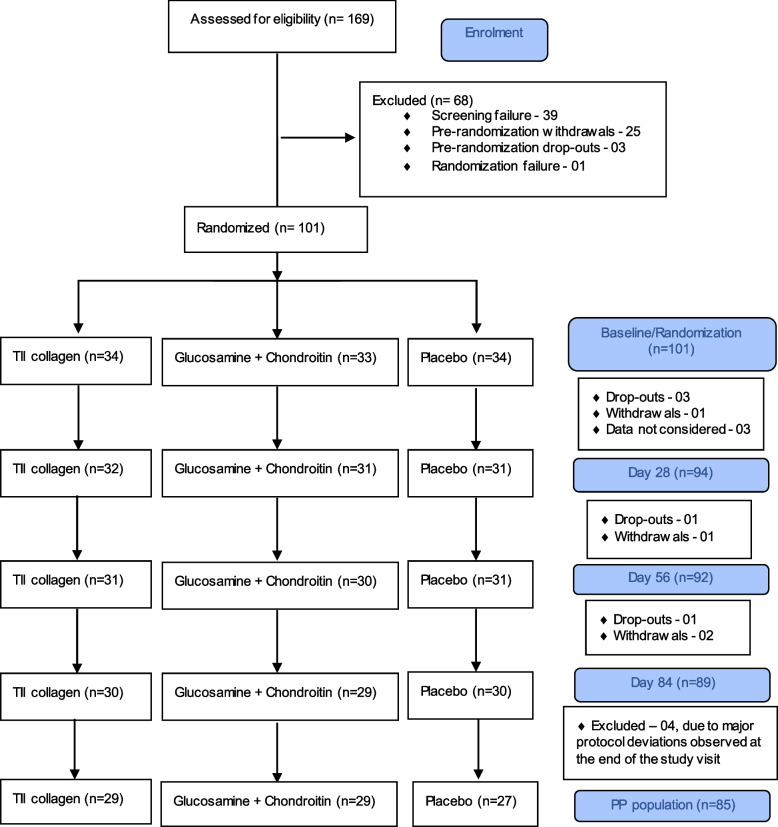
Participant disposition

One hundred and sixty-nine participants were screened for the study and a total of 101 participants were randomised into 3 study groups, with 34 participants in the TII collagen group, 33 in the G + C group, and 34 in the placebo group. Finally, 89 participants completed the study as provided in Fig. 1.

### Participant demographics and baseline characteristics

The participants were predominantly females (> 70%) with a mean age of 50.37 (range 40.00—64.00) years and comparable BMI (p = 0.6857). The other demographic and baseline characteristics for the randomised study population are given in Table 3.

**Table 3:** Demographic and baseline characteristics

Parameters	Statistics	TII collagen (N = 34)	G + C (N = 33)	Placebo (N = 34)	Total (N = 101)	p-value
**Age (years)**	Mean ± SD	50.94 ± 7.13	51.48 ± 6.44	48.71 ± 7.31	50.37 ± 7.01	0.2272
**BMI (kg/m**^**2**^**)**	Mean ± SD	24.79 ± 2.62	25.30 ± 3.03	25.24 ± 2.34	25.11 ± 2.66	0.6857
**Joint pain history (months)**	Mean ± SD	13.53 ± 5.98	13.52 ± 7.54	12.24 ± 6.94	13.09 ± 6.80	0.6721
**X-ray grade**	Grade-II, n (%)	20 (58.82%)	20 (60.61%)	20 (58.82%)	60 (59.41%)	0.9855
	Grade-III, n (%)	14 (41.18%)	13 (39.39%)	14 (41.18%)	41 (40.59%)	
**Index knee pain VAS score (mm)**	Mean ± SD	72.65 ± 9.63	70.30 ± 7.28	71.18 ± 8.80	71.39 ± 8.61	0.5338
**WOMAC total score**	Mean ± SD	86.06 ± 7.57	86.67 ± 7.21	86.45 ± 6.46	86.39 ± 7.03	0.9386
**WOMAC—Pain score**	Mean ± SD	14.38 ± 2.07	14.88 ± 1.87	14.50 ± 1.91	14.58 ± 1.95	0.5573
**WOMAC—Stiffness score**	Mean ± SD	5.91 ± 1.00	6.42 ± 0.90	6.26 ± 0.90	6.20 ± 0.95	0.0754
**WOMAC—physical function score**	Mean ± SD	65.76 ± 5.79	65.36 ± 5.99	65.47 ± 4.77	65.53 ± 5.48	0.9538
**EQ-5D – mobility score**	Mean ± SD	2.97 ± 0.63	3.15 ± 0.57	3.12 ± 0.73	3.08 ± 0.64	0.4749
**EQ-5D—self-care score**	Mean ± SD	2.74 ± 0.86	3.00 ± 0.66	2.97 ± 0.83	2.90 ± 0.79	0.3269
**EQ-5D—usual activities score**	Mean ± SD	3.09 ± 0.71	3.06 ± 0.79	3.35 ± 0.65	3.17 ± 0.72	0.1859
**EQ-5D—Pain or discomfort score**	Mean ± SD	3.24 ± 0.70	3.12 ± 0.82	3.32 ± 0.47	3.23 ± 0.68	0.4758
**EQ-5D—Anxiety or depression score**	Mean ± SD	2.79 ± 0.88	2.79 ± 0.89	3.09 ± 0.67	2.89 ± 0.82	0.2318
**EQ-5D—VAS score**	Mean ± SD	55.09 ± 16.00	51.82 ± 16.81	56.62 ± 13.69	54.53 ± 15.52	0.4387

Notes: *p*-values were calculated using ANOVA for continuous variables and Chi-Square/ Fisher Exact test for categorical variables

Abbreviation: *BMI* Body mass index, *G + C* Glucosamine hydrochloride and Chondroitin sulfate, *VAS* Visual analogue scale, *WOMAC* Western Ontario and McMaster Universities Osteoarthritis Index

### Effect of TII collagen on joint health

A significant reduction of the total scores from baseline was seen in all treatment groups (p < 0.05) at the end of week 4, 8, and 12. Compared with the placebo, the TII collagen group had a significantly reduced total score after 4 weeks, and a similar change was observed in the G + C group. Subsequently, after 12 weeks, the TII collagen and G + C groups showed a gradual and greater reduction in the total score. Furthermore, despite the substantially lower dose, the reduction in the score for the TII collagen group remained statistically and clinically comparable with G + C, as shown in Table 4.

**Table 4:** Change in WOMAC index total scores and specific domains in placebo, G + C, and TII collagen group (mITT population)

**Parameters**	**Week**	**TII collagen** **(N = 32)**	**G + C** **(N = 31)**	**Placebo** **(N = 31)**	**p-value**		
					Overall	TII collagen vs placebo	G + C vs placebo
**WOMAC- total score**	4	-15.28 ± 13.76	-17.94 ± 16.52	-5.87 ± 12.61	0.0036	0.0099	0.0015
	8	-22.75 ± 15.30	-25.19 ± 20.06	-10.52 ± 13.42	0.0012	0.0030	0.0007
	12	-32.47 ± 19.51	-33.74 ± 24.64	-13.84 ± 17.61	0.0002	0.0003	0.0003
**WOMAC- pain score**	4	-2.25 ± 3.01	-2.94 ± 3.35	-1.10 ± 2.75	-	-	0.0239
	8	-3.94 ± 2.61	-4.26 ± 3.95	-1.87 ± 2.95	0.0053	0.0049	0.0048
	12	-5.69 ± 3.66	-6.03 ± 4.72	-2.71 ± 3.95	0.0023	0.0024	0.0024
**WOMAC- stiffness score**	4	-1.00 ± 1.52	-1.42 ± 1.46	-0.23 ± 1.09	0.0030	0.0093	0.0013
	8	-1.53 ± 1.70	-1.97 ± 1.62	-0.61 ± 1.38	0.0014	0.0024	0.0012
	12	-2.19 ± 1.75	-2.71 ± 2.05	-0.84 ± 1.27	< 0.0001	0.0002	< 0.0001
**WOMAC- physical function score**	4	-12.03 ± 10.16	-13.58 ± 12.53	-4.55 ± 9.35	0.0028	0.0068	0.0014
	8	-17.28 ± 12.00	-18.97 ± 15.62	-8.03 ± 10.19	0.0017	0.0042	0.0010
	12	-24.59 ± 15.00	-25.00 ± 18.90	-10.29 ± 13.07	0.0002	0.0003	0.0002

Notes: value presented as Mean ± SD

p-values were calculated for the absolute change in WOMAC scores compared to placebo using ANCOVA with treatment and visit as factors and baseline as a covariate

Abbreviation: *G + C* Glucosamine hydrochloride and Chondroitin sulfate

### Effect of TII collagen on joint pain

The within-group analysis demonstrated a statistically significant difference in each group for all study visits compared with the baseline, with the extent of reduction increased with the longer treatment duration. However, the intergroup analysis showed that while the absolute change in the WOMAC-P scores for the G + C group was statistically significant compared to the placebo (p < 0.05) for all study visits, the difference between the TII collagen and placebo group was only significant at the end of week 8 and 12. No statistically significant difference was observed for the absolute change in the scores between TII collagen and G + C groups on all visits, which shows that TII collagen was as effective as the positive control in relieving joint pain (Table 4).

### Effect of TII collagen on stiffness

For the WOMAC-S subscale, the within-group analysis demonstrated a statistically significant difference between the TII collagen and the G + C group for all study visits compared to the baseline, with an increase in the reduction from week 4 to 12. When changes in scores in both the TII collagen and G + C groups were compared with those in the placebo, each group showed a statistically significant difference during all treatment visits. Furthermore, the change in the scores was comparable between the TII collagen and the G + C groups on all follow-up visits (Table 4).

### Effect of TII collagen on physical function

Within-group analysis for the treatment groups demonstrated a statistically significant difference in WOMAC-PF subscale scores for each group at all study visits compared to the baseline, with a persistent increase in the score reduction from week 4 onwards. Again, when the TII collagen and G + C group changes in scores were compared with the placebo, each group showed a statistically significant difference during all treatment visits. Furthermore, no significant difference was observed in the change in the physical function scores between TII collagen and the G + C groups on all follow-up visits (Table 4), which shows a similar effect of both treatments on the physical function of the knee joint.

### Effect of TII collagen on quality of life

The week 12 assessment was completed by 89 participants, as given in Table 5. After a treatment period of 12 weeks, a significant difference was seen within all quality of life domains and the VAS score compared to the baseline. Furthermore, the absolute change within the TII collagen was statistically significant compared to that in the placebo for 4 of the 5 domains and the VAS score, except for the "anxiety or depression" domain. The same trend was observed within the G + C group scores; however, TII collagen saw a more significant change in the "Usual activities" than in the G + C group compared to placebo (*p* = 0.0156 vs *p* = 0.1997, respectively).

**Table 5:** Change in EQ-5D-5L scores at week 12 from baseline (mITT population)

EQ-5D-5L domains	TII collagen (N = 30)	G + C (N = 29)	Placebo (N = 30)	p-value	
				TII collagen vs Placebo	G + C vs Placebo
**EQ-5D—mobility**	-1.00 ± 0.74	-1.21 ± 0.73	-0.73 ± 0.98	0.0255	0.0179
**EQ-5D—self-care**	-0.90 ± 0.84	-1.00 ± 0.89	-0.47 ± 0.97	0.0112	0.0340
**EQ-5D—usual activities**	-1.17 ± 0.87	-0.93 ± 0.92	-0.83 ± 1.02	0.0156	-
**EQ-5D—pain or discomfort**	-1.13 ± 0.68	-0.97 ± 1.02	-0.60 ± 0.72	0.0020	0.0117
**EQ-5D—anxiety or depression**	-0.90 ± 0.76	-1.00 ± 1.07	-0.93 ± 1.17	-	-
**EQ-5D—VAS Score**	17.73 ± 13.78	18.97 ± 15.55	4.50 ± 13.73	0.0003	0.0009

Notes: values presented as Mean ± SD

*p*-value was calculated using ANCOVA with treatment and visit as a factor and baseline as covariate vs placebo

Abbreviation: *G + C* Glucosamine hydrochloride and Chondroitin sulfate

### Use of rescue medication

There was no statistical difference between the proportion of participants who consumed rescue medication throughout the study period (p > 0.05).

### Safety evaluations

No statistically significant difference between the groups was observed in any safety parameters (vitals and biomarkers) during baseline (p > 0.05). Both vitals and biomarkers did not change beyond the normal range over the study period.

All but one adverse event reported in the study were mild (boils on back and hand (*n* = 1, TII), itching (*n* = 1, placebo), reduced appetite (*n* = 1, placebo), increased SGPT-grade I (*n* = 1, placebo)) and resolved completely. None of the AEs observed during the study were related to the study products. The serious adverse event observed in the study was reported for one participant diagnosed with COVID-19 and was unrelated to the study product.

## Discussion

The main finding of this study suggest that TII collagen is considerably effective in improving the overall symptoms of OA, as depicted by a statistically significant reduction in WOMAC total scores by the end of week 4, which progressively increased over longer periods of dosing. Furthermore, the reduction in mean WOMAC total scores in the TII collagen group on week 4, 8, and 12 was comparable to that in the G + C group with no statistically significant difference (*p* > 0.05), thereby demonstrating the non-inferiority of TII collagen compared to the active comparator, G + C.

Also, in this study, TII collagen consumption significantly reduced pain, stiffness, and physical function associated with OA compared to baseline and placebo. The efficacy increased throughout the study period, with maximal efficacy observed at the end of the study (week 12). Furthermore, the absolute change in all subscale scores was comparable with G + C at all time points (*p *> 0.05), reiterating the non-inferiority of TII collagen to G + C in eliciting significant benefits in OA-associated pain, stiffness, and physical function.

The use of type II collagen in combination with other products such as manganese ascorbate has also shown statistical improvements in the management and treatment of OA compared to placebo [23]. Another study conducted to evaluate the efficacy of undenatured type II collagen observed that the WOMAC score for the undenatured type II collagen was reduced by 33% from baseline. In comparison, it was reduced by only 14% in participants in the G + C group after a treatment period of 90 days, the reduction between both groups being statistically significant [14]. Based on these studies, it can be concluded that undenatured type II collagen has a proven efficacy toward OA symptomatic relief either equivalent to or better than G + C in some cases, as was seen due to the statistically comparable reduction in scores. Additionally, it is effective at low doses and hence more potent than G + C. The longer treatment duration for the current study may have reflected similar results as observed in the two studies mentioned above.

In addition to the symptomatic presentation of OA, the joint impairment associated with knee pain in OA also negatively impacts a person's quality of life [24, 25]. The limitations in the activity that arises due to OA progression impact the psychological well-being and social life of individuals, thus reducing their quality of life. This is why assessing the quality of life in patients with OA is crucial to comprehend the full impact of the disease and any improvements achieved through various treatments [26].

To evaluate the impact of TII collagen on the participants' quality of life, the EQ-5D-5L questionnaire was used in this study. At the end of the 12 weeks treatment period, each domain in the EQ-5D-5L showed a statistically significant improvement in the quality of life in the TII collagen group compared with the placebo, except for the "anxiety or depression" domain. Again, the scores of the TII collagen group of 40 mg undenatured type II collagen and G + C group of glucosamine hydrochloride 1500 mg combined with chondroitin sulfate 1200 mg were comparable for all domains at the end of the treatment period, even though the reduction in mean "usual activities" scores in the TII collagen group was numerically greater than that of the G + C group. Furthermore, safety assessments conducted on the tested dose did not reveal any safety or tolerability concerns throughout the treatment duration. Even the adverse events recorded during the study did not relate to the study products.

Joint wear and tear have been acknowledged to cause significant disability and isolation in the ageing population [27]. Numerous nutraceuticals have been developed over the past decades to combat joint impairment, primarily due to OA. This was mainly done to avoid invasive techniques and therapies such as NSAIDs that would lead to significant adverse effects. Some compounds for joint health improvement include collagen peptide, chondroitin sulfate, glucosamine sulfate, fish oil, Boswellia, green tea, ginger, and rosehip extracts [28].

The products that have been clinically proven to be effective in OA management include a combination of glucosamine and chondroitin sulfate [12, 29–31], Boswellia, curcumin, pycnogenol, methylsulfonylmethane, and undenatured type II collagen [32]. Out of these products, undenatured type II collagen is one of the few products that provides a significant effect at a relatively low dose [14, 15]. The treatment duration for the OA trials varies between 4 weeks and 3 years. The dosage is low only in undenatured type II collagen (40 mg/day) and pycnogenol (50 mg/day), while it was as high as 100 mg/day for *Boswellia serrata,* 800 mg to 1200 mg/day for chondroitin sulfate, 1500 mg/day for glucosamine hydrochloride or glucosamine sulfate and 10 g/day for collagen hydrolysate [33]. The combination of glucosamine hydrochloride & chondroitin sulfate has been utilised worldwide to treat OA, with studies still underway to evaluate its further efficacy. Similar to many studies, the current study demonstrated that TII collagen is safe for use and has an effective impact on the symptomatic effects of knee impairment associated with OA and on the quality of life of the participants. Additionally, TII collagen also had a higher potency than G + C. The greatest improvement in all assessments was observed at the end of the study (week 12); thus, it can be postulated that further symptomatic and quality-of-life benefits would emanate with even longer chronic dosing periods. The study has some limitations; firstly, the sample size was small, and secondly, the treatment duration was shorter. Therefore, a well-powered and longer-duration study to explore the chondroprotective effect of TII collagen is warranted.

## Conclusion

This study demonstrated the statistically significant effect of TII collagen in improving joint health and quality of life in individuals with OA at a small dosage of 40 mg compared with the positive control combination of glucosamine hydrochloride and chondroitin sulfate at a dose of 2700 mg.

## Data Availability

The data generated and analysed during the study are available on reasonable request from the corresponding author, with due permission from the sponsor.

## Abbreviations

ALP: Alkaline phosphatase
ALT: Alanine aminotransferase
ANCOVA: Analysis of covariance
ANOVA: Analysis of variance
AST: Aspartate aminotransferase
BMI: Body mass index
DBP: Diastolic blood pressure
FBG: Fasting blood glucose
G + C: Glucosamine hydrochloride + chondroitin sulfate
IP: Investigational product
mITT: Modified intention-to-treat
NSAIDs: Nonsteroidal anti-inflammatory drugs
OA: Osteoarthritis
SBP: Systolic blood pressure
TII: Type II
VAS: Visual analogue scale
WOMAC: Western Ontario and McMaster Universities Osteoarthritis Index

## References

[1] Hsu H, Siwiec RM. (2022) Knee Osteoarthritis. In: StatPearls. Treasure Island (FL), StatPearls Publishing. Available from: https://www.ncbi.nlm.nih.gov/books/NBK507884/.

[2] Cui A, Li H, Wang D, Zhong J, Chen Y, Lu H (2020). Global, regional prevalence, incidence and risk factors of knee osteoarthritis in population-based studies. EClinicalMedicine.

[3] MarketsandMarkets (2020) Osteoarthritis Therapeutics Market - Global Forecast to 2025. https://www.marketsandmarkets.com/Market-Reports/osteoarthritis-therapeutics-market-209565994.html?gclid=CjwKCAjw2bmLBhBREiwAZ6ugo5Mqec4ajgZ8UxP4N6eR59eIIuOockof56d8Qu1ivJEQxmLS79_PVxoCKksQAvD_BwE. Accessed October 19, 2021

[4] Osama A, Zakir H, Abdel S, Yegappan K (2018) Treatment modalities for hip and knee osteoarthritis: A systematic review of safety. J Orthop Surg (Hong Kong) 26(3):2309499018808669 10.1177/2309499018808669 30415598

[5] Kamble S, Patil A, Shinde S, Ankush H (2021). A review on current nutraceuticals in the management of osteoarthritis. Int J Hortic Food Sci.

[6] Desai A, Shendge NP, Anand SS (2021). Evidence-based nutraceuticals for osteoarthritis: A review. Int J Orthop Sci.

[7] Gupta RC, Lall R, Srivastava A (eds) (2021) Nutraceuticals - efficacy, safety and toxicity, 2nd edn. Academic Press, p 1396. Published: January 27, 2021. Imprint: Academic Press. Hardcover ISBN: 9780128210383. https://www.elsevier.com/books/nutraceuticals/gupta/978-0-12-821038-3

[8] Henrotin Y, Mobasheri A, Marty M (2012). Is there any scientific evidence for the use of glucosamine in the management of human osteoarthritis?. Arthritis Res Ther.

[9] Kirkham S, Samarasinghe R (2009). Review article: Glucosamine. J Orthop Surg (Hong Kong).

[10] Jackson C, Plaas A, Sandy J (2010). The human pharmacokinetics of oral ingestion of glucosamine and chondroitin sulfate taken separately or in combination. Osteoarthr Cartil.

[11] Jerosch J (2011). Effects of Glucosamine and Chondroitin Sulfate on Cartilage Metabolism in OA: Outlook on Other Nutrient Partners Especially Omega-3 Fatty Acids. Int J Rheumatol.

[12] Lomonte ABV, Mendonça JA, de Brandão G, C, Castro ML,  (2018). Multicenter, randomised, double-blind clinical trial to evaluate efficacy and safety of combined glucosamine sulfate and chondroitin sulfate capsules for treating knee osteoarthritis. Adv Rheumatol (London, England).

[13] Qiu GX, Gao SN, Giacovelli G, Rovati L, Setnikar I (1998). Efficacy and safety of glucosamine sulfate versus ibuprofen in patients with knee osteoarthritis. Arzneimittelforschung.

[14] Crowley DDC, Lau FFC, Sharma P (2009). Safety and efficacy of undenatured type II collagen in the treatment of osteoarthritis of the knee: a clinical trial. Int J Med Sci.

[15] Lugo JP, Saiyed ZM, Lane NE (2016). Efficacy and tolerability of an undenatured type II collagen supplement in modulating knee osteoarthritis symptoms: A multicenter randomised, double-blind, placebo-controlled study. Nutr J.

[16] Orhan C, Juturu V, Sahin E (2021). Undenatured Type II Collagen Ameliorates Inflammatory Responses and Articular Cartilage Damage in the Rat Model of Osteoarthritis. Front Vet Sci.

[17] Wu TT, Zhu MH, Wang GP, Wang HG (2020). Improving effect of Native CT-II on the monoiodoacetic acid-Induced Osteoarthritis in rats. China Pharmaceuticals.

[18] Lugo J, Saiyed Z, Lau F (2013). Undenatured type II collagen (UC-II®) for joint support: a randomised, double-blind, placebo-controlled study in healthy volunteers. J Int Soc Sports Nutr.

[19] Chopra A, Lavin P, Patwardhan B, Chitre D (2004). A 32-week randomised, placebo-controlled clinical evaluation of RA-11, an Ayurvedic drug, on osteoarthritis of the knees. J Clin Rheumatol.

[20] Chopra A, Lavin P, Patwardhan B, Chitre D (2000). Randomized double-blind trial of an ayurvedic plant derived formulation for treatment of rheumatoid arthritis. J Rheumatol.

[21] Bilbao A, García-Pérez L, Arenaza J (2018). Psychometric properties of the EQ-5D-5L in patients with hip or knee osteoarthritis: reliability, validity and responsiveness. Qual life Res.

[22] Braham R, Dawson B, Goodman C (2003). The effect of glucosamine supplementation on people experiencing regular knee pain. Br J Sports Med.

[23] Das A, Hammad T (2000). Efficacy of a combination of FCHG49 glucosamine hydrochloride, TRH122 low molecular weight sodium chondroitin sulfate and manganese ascorbate in the management of knee osteoarthritis. Osteoarthr Cartil.

[24] Lespasio MJ, Piuzzi NS, Husni ME, Muschler GF, Guarino A, Mont MA (2017). Knee Osteoarthritis: A Primer Perm J.

[25] Salaffi F, Carotti M, Stancati A, Grassi W (2005). Health-related quality of life in older adults with symptomatic hip and knee osteoarthritis: a comparison with matched healthy controls. Aging Clin Exp Res.

[26] Vitaloni M, Bemden AB, Contreras RS (2019). Global management of patients with knee osteoarthritis begins with quality of life assessment: a systematic review. BMC Musculoskelet Disord.

[27] Hughes SL, Dunlop D, Edelman P, Chang RW, Singer RH (1994). Impact of joint impairment on longitudinal disability in elderly persons. J Gerontol.

[28] Vaishya R, Agarwal A, Shah A, Vijay V, Vaish A (2018). Current status of top 10 nutraceuticals used for Knee Osteoarthritis in India. J Clin Orthop trauma.

[29] Fransen M, Agaliotis M, Nairn L (2015). Glucosamine and chondroitin for knee osteoarthritis: a double-blind randomised placebo-controlled clinical trial evaluating single and combination regimens. Ann Rheum Dis.

[30] Hochberg MC, Martel-Pelletier J, Monfort J (2016). Combined chondroitin sulfate and glucosamine for painful knee osteoarthritis: a multicentre, randomised, double-blind, non-inferiority trial versus celecoxib. Ann Rheum Dis.

[31] Provenza JR, Shinjo SK, Silva JM, Peron CR, Rocha FA (2015). Combined glucosamine and chondroitin sulfate, once or three times daily, provides clinically relevant analgesia in knee osteoarthritis. Clin Rheumatol.

[32] Liu X, Machado GGC, Eyles JPJ, Ravi V, Hunter DJD (2018). Dietary supplements for treating osteoarthritis: a systematic review and meta-analysis. Br J Sports Med.

[33] Liu X, Eyles J, McLachlan AJA, Mobasheri A (2018). Which supplements can I recommend to my osteoarthritis patients?. Rheumatology (Oxford)..

